# The Role of Surgery in Spinal Intradural Metastases from Renal Cell Carcinoma: A Literature Review

**DOI:** 10.3390/cancers14061595

**Published:** 2022-03-21

**Authors:** Sergio Corvino, Giuseppe Mariniello, Domenico Solari, Jacopo Berardinelli, Francesco Maiuri

**Affiliations:** Department of Neurosciences, Reproductive and Odontostomatological Sciences, Neurosurgical Clinic, School of Medicine, University of Naples “Federico II”, 80131 Naples, Italy; giumarin@unina.it (G.M.); domenico.solari@unina.it (D.S.); iacopobe96@gmail.com (J.B.); frmaiuri@unina.it (F.M.)

**Keywords:** metastatic clear renal cell carcinoma, spinal metastases, intradural extramedullary, intramedullary metastases

## Abstract

**Simple Summary:**

Renal cell carcinoma is a highly metastatic tumor, mainly to the lungs (50%), bone (49%), lymph-nodes (6–32%), liver (8%), and brain (3%). A wide and accurate literature review has disclosed only 51 cases of intradural spinal metastasis from sporadic renal cell carcinoma, of which 32 at intramedullary and 19 at extramedullary localizations. Once detected, they represent a sign of advanced disease and often lead to rapidly progressive neurological deficits. Because of these few reported data, there are no defined guidelines of treatment and the decision making in the choice of the best strategy should consider the curative, functional and palliative aspects, accordingly the management should be tailored for each patient. The options include surgery, radiotherapy, and chemotherapy, which can be performed in isolation or various combinations at the discretion of each institution. We discuss the role of surgery in the management of spinal intradural metastases from renal cell carcinoma.

**Abstract:**

Background: Due to the few reported cases of spinal intradural metastases from renal cell carcinoma (RCC), there is no unanimous consensus on the best treatment strategy, including the role of surgery. Methods: A wide and accurate literature review up to January 2022 has disclosed only 51 cases of spinal intradural metastases from RCC. Patients with extramedullary (19) and those with intramedullary (32) localization have been separately considered and compared. Demographics, clinical, pathological, management, and outcome features have been analyzed. Results: Extramedullary lesions more frequently showed the involvement of the lumbar spine, low back pain, and solitary metastasis at diagnosis. Conversely, the intramedullary lesions were most often detected in association with multiple localizations of disease, mainly in the brain. Surgery resulted in improvement of clinical symptoms in both groups. Conclusion: Several factors affect the prognosis of metastatic RCC. The surgical removal of spinal metastases resulted in pain relief and the arresting of neurological deficit progression, improving the quality of life and overall survival of the patient. Considering the relative radioresistant nature of the RCC, the surgical treatment of the metastasis is a valid option even if it is subtotal, with a consequent increased risk of recurrence, and/or a nerve root should be sacrificed.

## 1. Introduction

Renal cell carcinoma (RCC) accounts for nearly 15,000 deaths per year in the United States [1], with a poor overall survival rate at 5 years despite the advances in radiologic imaging resulting in an early diagnosis, and in the management [2,3]. Metastases occur in 35% of patients with RCC and are synchronous in 15% and metachronous in the remaining 20% of cases [4]; the most common involved location is the lung, followed by the bone and lymph-nodes [5]. To the best of our knowledge, only 51 cases of spinal intradural metastases (19 extramedullary and 32 intramedullary) from sporadic renal cell carcinoma are reported in the literature.

Metastatic lesions involving the spinal intradural space often lead to rapidly progressive neurological deficits, resulting in severe quality of life (QoL) worsening. Because of the small sample of reported cases, there is no unanimous consensus about the best strategy for treatment. The management options include surgery, radiation, and chemotherapy, administered in isolated or various combined manner, at discretion of each institution.

We report a wide and accurate literature review, including a case recently treated at our institution [6], and we discuss the role of surgery in the management of both intra- and extramedullary intradural spinal metastases from RCC.

## 2. Methods

A Medline search up to January 2022 in PubMed online electronic database was made using the following key phrases: “intradural spinal metastasis and clear renal cell carcinoma”, “intradural extramedullary metastasis and clear renal cell carcinoma”, and “intramedullary spinal metastasis and clear renal cell carcinoma”. The inclusion criteria were surgical series, reviews, and case reports in English language, as well as papers written in other languages, but including the abstract in English. All reported cases of spinal intradural metastasis, either extra- or intramedullary, from renal cell carcinoma, meeting the inclusion criteria, were enrolled.

Patients with spinal intradural extramedullary and those with intramedullary metastases were separately considered and, after, compared.

The analyzed factors were patient sex and age at diagnosis of spinal intradural metastasis, latency between the diagnoses of renal cell carcinoma and spinal intradural metastasis, spinal level involved, presence and location of systemic metastases, presenting symptoms, management and clinical outcome of the spinal metastasis, and overall survival.

Characteristics between groups and subgroups of categorial data were compared using Pearson’s chi-square test and Fisher’s exact test. Survival curves were illustrated by Kaplan-Meier method with univariate survival analysis. P values of less than 0.05 were considered significant.

## 3. Results

### 3.1. Literature Review of Intradural Extramedullary (IDEM) Spinal Metastasis

To the best of our knowledge, only 19 cases of spinal intradural extramedullary metastasis from renal cell carcinoma, including our own [6], have been reported in the literature [6,7,8,9,10,11,12,13,14,15,16,17,18,19,20,21,22,23,24] (Table 1).

Among them, 13 (68%) were males and 6 (32%) were females, with an average age at diagnosis of spinal metastasis of 61.84 ± 14.27 SD years (range from 36 to 84 yrs). The mean interval from the diagnosis of the primary tumor and of spinal intradural metastasis was 61.88 ± 65.65 SD months, including 5 cases (26%) of synchronous and 14 cases (74%) of metachronous metastasis. The spinal segments were involved as follows: 14 (75%) cases were lumbar, 2 (10%) thoracic, 1 (5%) cervical, and 2 (10%) thoraco-lumbar. The most common presenting symptom was low back pain (LBP) (13/19, 68%), followed by radicular symptoms (9/19, 47%), leg weakness (7/19, 37%), and urinary incontinence (7/19, 37%); one case (5%) was asymptomatic and was discovered during radiologic investigation for other pathologies. Other secondary localizations of disease were detected in 10 (53%) among 19 cases, and were at lung (6/10), bone (3/10), brain (1/10), lymph-nodes (1/10), and leptomeninges (1/10); in eight cases (42%) the IDEM was the unique metastasis; finally, in one case this date was not specified. All patients but one (95%), who refused, underwent surgical resection of spinal metastasis; during the surgery, the involved nerve root was preserved in 7 and sacrified in 8 among the 15 cases in which this date was reported. Adjuvant treatments were administered in 13 cases and consisted of radiotherapy (RT) alone in 8, chemotherapy alone in 2, a combination of both in the other 2, and finally interferon in the last 2 patients. Chemotherapy as the only treatment was administered to the unique patient who refused the surgery. Post-treatment clinical symptoms improved in 15 (80%) among 19 patients, were stable in 2 (10%), and worsened in the remaining 2 (10%).

The follow-up, ranging from 1 to 108 months (mean 25.5 ± 25.6 SD months) among 15 cases in which it was reported, showed 12 patients (80%) alive and 3 (20%) died for progression of primary disease at last follow-up.

All these data are summarized in Table 2.

### 3.2. Literature Review of Intramedullary Spinal Cord Metastasis (ISCMs)

The literature review has disclosed 32 cases [25,26,27,28,29,30,31,32,33,34,35,36,37,38,39,40,41,42,43,44,45,46,47,48] of intramedullary spinal cord metastasis from renal cell carcinoma (Table 3). Among them, 25 (78%) were males and 7 (22%) were females, with a median age of 55.96 ± 10.89 SD years (range from 37 to 78 yrs). The interval between the diagnosis of the primary disease and the intramedullary metastasis ranged from 0 to 180 months (median 28.76 ± 45.31 SD months) as follows: to note that in nine cases (28%) the spinal metastasis was diagnosed simultaneously with the renal cell carcinoma; in the remaining cases (72%) it was metachronous. In about half of the cases (14/32, 44%), the cervical segment of the spine was involved, followed by the thoracic (13/32, 41%) and the lumbar ones (3/32, 9%); the remaining two cases (6%) occurred at the thoraco-lumbar junction. Concerning the presenting clinical symptoms, limb weakness was referred in 37.5% of cases (12/32), followed by motility disturbance (9/32, 28%), spinal pain (8/32, 25%) and urinary disfunction (6/32, 19%); 2 (6%) cases of Brown-Sequard syndrome were also present.

Secondary localizations of disease were known in 30 cases (94%) and including lung (15/30, 50%), brain (12/30, 40%), bone (6/30, 20%), lymph-nodes (5/30, 17%), adrenal gland (3/30, 10%), and liver (2/30, 7%); in 5 cases (17%), the intramedullary spinal compartment was the unique site of metastasis; finally in 2 cases this date was not specified.

Twenty-one (66%) patients underwent surgical resection; radiotherapy was administered to 19 patients (59%) (as adjuvant in 10, combined with drugs in 6, as single treatment in 1 and associated with SRS in 1); two cases refused surgical treatment and were treated by RT alone (1) and by medical therapy (1).

Post-treatment clinical conditions were not specified in 5 cases; among the remaining 27, 16 (59%) reported an improvement in symptoms, 9 (34%) were stable, and finally, only two patients (7%) referred a worsening.

Reported overall survival (in 28 among 32 patients) ranged from 1 to 65 months (mean 13.15 ± 17.09 SD) and showed 14 (50%) dead cases and 14 (50%) alive at the last follow-up.

All these data are summarized in Table 2.

### 3.3. Intradural Extramedullary and Intramedullary Spinal Cord Metastases from RCC at Mirror

The comparative analysis between intradural extramedullary and intramedullary spinal metastases from RCC (Table 2) showed a similar distribution in terms of sex and age.

The detection of spinal metastasis was metachronous in about ¾ of cases in both groups; nevertheless, the latency was longer in extramedullary than in intramedullary metastasis, mean 61.88 ± 65.66 months versus 28.76 ± 45.31 months, respectively.

Concerning the spinal levels involvement, the lumbar tract was most often affected in the IDEM metastasis (*p* < 0.00001), while the cervical one was the most affected in intramedullary lesions (*p* = 0.0035).

Spinal pain was referred as presenting symptom mainly in patients with extramedullary lesions (68% vs. 25%, *p* = 0.002) and it was mostly low back pain.

Intradural extramedullary compartment was often the unique site of metastasis (42%) compared to the intramedullary (17%) which was more often associated to other secondary localizations of disease (*p* = 0.036), mainly lung (50%), and brain (40%) (*p* = 0.008).

Surgery was performed in all but one case of intradural extramedullary (95%) and in 11 among 32 patients (35%) with intramedullary metastasis (*p* < 0.00001), and it was associated in both cases with postoperative clinical symptom improvement in most patients (Table 4 and Table 5).

**Figure 1:**
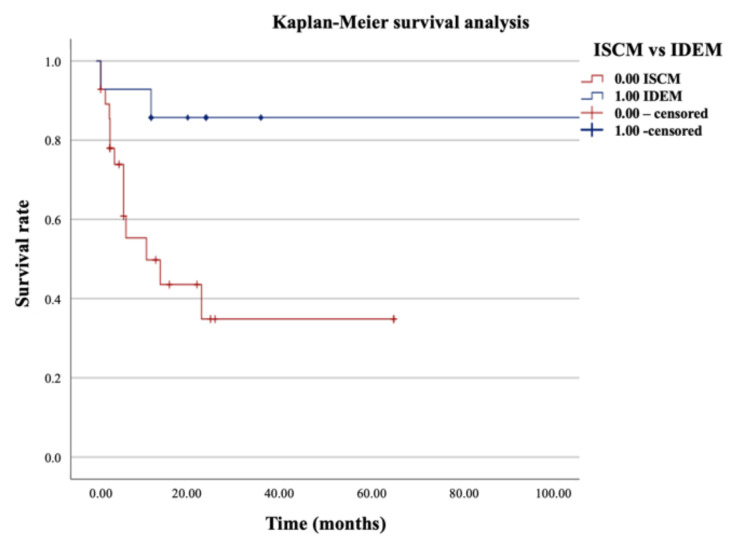
Kaplan-Meyer survival analysis between Intradural extramedullary (IDEM) and intramedullary (ISCMs) spinal metastases from RCC.

Finally, the overall survival was better in IDEM compared to the intramedullary metastases, with 80% versus 50% of patients alive at last follow-up and a mean survival of 25.5 ± 25.6 months versus 13.15 ± 17.09 months (*p* = 0.011), respectively (Figure 1).

## 4. Discussion

Spinal intradural metastases are extremely rare and may also occur after many years from the diagnosis of the primary malignancy, posing problems in differential diagnosis and management.

Metastases to the spinal intradural space can occur through the following different ways: hematogenous dissemination, venous dissemination, via perineural lymphatics, subarachnoid space, and through direct invasion from near anatomical structures [9,15,49].

Because of the few reported data concerning spinal intradural, both extra and intramedullary, metastasis from RCC, resulting from the rarity of this pathological entity, there is not a unanimous consensus about their best management; the armamentarium at disposition includes surgery, radiotherapy, and chemotherapy, performed in an isolated manner or in various associations.

Several factors must be considered before choosing the best option of treatment. First, the radio and/or chemo-resistant nature of the primary tumor, the expectancy of life of the patient, its performance status, and the presence of comorbidities that could contraindicate some procedures. Keeping in mind the curative, functional, and palliative aspects; accordingly, the management should be tailored for each patient.

Surgery represents the gold standard of treatment for spinal metastases with acute onset of neurological symptoms, with the aim of arresting the decline of neurological functions, improving clinical symptoms (neurological deficits and/or pain), and preventing new-onset, potentially irreversible neurological deficits through the decompression of neural structures. RCC is a highly vascularized tumor with a tendency to bleed as follows: a sudden bleeding in a non-expandible spinal canal leads to a mass effect on the spinal cord or nerve roots, resulting in a potentially irreversible neurological deficit.

Potential complications include approach-related morbidities, intraoperative hemorrhage, CSF leak, thromboembolism, and wound infections. Their occurrence risk may be decreased with careful preoperative planning which considers the goals of treatment in terms of subtotal or gross total tumor resection, nerve root preservation or sacrifice, based on life expectancy and functional outcome, a meticulous surgical technique, and adherence to medical guidelines supporting their prevention.

Indeed, intra- and postoperative complications have not been reported in all the described cases in our review.

The choice of the surgical approach—laminoplasty, monolateral, or bilateral laminectomy—depends on the localization of the tumor in the vertebral canal, its pattern of growth, and its relationship with the spinal cord and nerve roots; the important thing is to gain adequate exposure and access to the poles of the tumor, which can be verified by ultrasound before the dura mater opening. A CO_2_ laser could be useful to reduce mechanical stress. Some authors performed the tumor resection en bloc, others in fragments after internal debulking. Care should be taken to avoid the spread of tumor fragments in the subarachnoid space.

The occurrence risk of neurological morbidity can be decreased with the help of intraoperative monitoring (IOM); nerve root damage leads to variable consequences according to the involved spinal segment, from diaphragmatic paralysis for upper cervical roots to ambulation impairment for lumbar nerve root involvement. Although nerve root preservation would be desirable, it is not always achievable; in our review, the nerve root was preserved in 7 and sacrified in 8 patients.

Intraoperative hemorrhage is a serious potential complication, mainly for this highly vascularized tumor; therefore, an accurate preoperative radiological evaluation and careful tumor dissection are mandatory. CSF leak could lead to longer operation time and hospital-stay, as well as other complications such as meningitis, intracranial hypotension, cutaneous fistula; thus, a watertight closure of the dura should be performed with auto-allograft materials or sealants and bedrest for 2–7 days would be advisable.

An early and prompt diagnosis is crucial for the outcome, being the ability to recover a neurological deficit related to the duration and entity of compression; in fact, the postoperative improvement in lower-extremity function is more likely when preoperative impairment is mild [50,51,52].

The role of radiotherapy in metastatic RCC is controversial [53] and is underexplored for the following two reasons: first because in-vitro studies have indicated radio-resistance and second because data from older clinical trial have showed high rates of radiotherapy-related deaths [54,55]. Radiation therapy is less invasive than surgery and can stop tumor and neurological deficit progression; nevertheless, is reserved for patients with smaller metastatic lesions and radiosensitive tumors.

### 4.1. Intradural Extramedullary (IDEM) Spinal Metastases

Several primary malignancies may metastasize to the intradural extramedullary space, including the lung, kidney, thyroid, prostate, and bone. The literature review has disclosed only the following 19 cases of IDEM metastasis from sporadic renal cell carcinoma (Table 1): spinal metastases were most commonly metachronous (74%), located in the spinal lumbar segment (75%), and isolated (42%).

The presence of intradural extramedullary metastasis is a sign of advanced disease and it is associated with decreased overall survival with a poor prognosis; accordingly, the goal of treatment—surgical, radiation therapy, or both—is often palliative to improve the quality of life.

Most of patients with intradural metastasis to the cauda equina from renal cell carcinoma undergone an early diagnosis and an appropriate and timely surgical resection, experimented immediate improvement of clinical symptoms [15], also after a simple debulking [12,16,19]. Nevertheless, very often, due to the close adherence of the lesion to a nerve root and the lack of a clear cleavage plane, a nerve root transection [19] or a subtotal resection of the lesion is necessary, with the possible consequence of neurological deficit new-onset and increased recurrence rate, respectively.

In our review, all but one case (who refused) (95%) underwent surgery, which involved nerve root preservation in seven and sacrified in eight patients, whereas adjuvant treatments were administered in thirteen cases (7 RT alone, 2 CHT alone, 2 IFN alone, 2 RT + CHT). Clinical conditions resulted in the improvement in most of the cases (80%).

According to the literature, local treatments for metastases from RCC, such as metastasectomy, showed a benefit in terms of overall survival (OS) and cancer-specific survival (CSS) [56,57,58,59], whereas RT has been shown to provide improvement in clinical symptoms and local control in RCC based on the delivered dose [60]. Furthermore, we must consider the relatively radioresistant nature of the renal cell carcinoma [61,62]. Our data agree with the literature, as shown in Table 4 as follows: clinical symptoms improved in all cases that had undergone surgery alone and in seven among eight that underwent adjuvant radiotherapy.

Besides, the surgery is not only indicated to reduce mass effect symptoms and prevent progression of neurological deficits, but also to define the nature of the underlying disease, because metastasis may be detected before the diagnosis of primary disease, or to define the nature of the metastasis that may occur several years after primary disease.

### 4.2. Intramedullary Spinal Cord Metastases (ISCMs)

Intramedullary spinal cord lesions are rare [63]; among them, metastases represent 4–8.5% of all central nervous system metastases; nevertheless, with the improved overall survival rates thanks to more effective treatments of primary malignancy and the early diagnosis of spinal metastasis thanks to the improvement in diagnostic techniques and protocols, their incidence is increasing.

Intramedullary spinal cord metastases mainly come from lung and breast cancers, less frequently from melanoma, thyroid, colorectal, ovarian, and renal cell carcinoma [64]. To the best of our knowledge, only 32 cases of ISCMs from renal cell carcinoma have been reported in the literature (Table 2).

In our review, most of the lesions were metachronous (72%), located in the cervical spine (44%), and associated with pulmonary (50%) and brain metastasis (40%) (*p* = 0.008) at diagnosis (Table 3). The frequent detection of spinal metastasis, associated with other secondary localizations of disease at diagnosis, represents a sign of advanced disease which, together with histological findings, life expectancy, quality of life, and neurological function affects the therapeutic iter.

Steroid drugs are administered to all patients at the onset of clinical symptoms with the aim of palliation.

Results from our review showed stationarity or improvement of clinical symptoms in all cases treated by surgery or radiotherapy, and only one case of worsening after surgery and adjuvant RT; nevertheless, the mean overall survival rate was better in patients who had undergone surgery than in those who had undergone radiotherapy alone or with adjuvant (Table 5).

Surgery represents the second choice of treatment after initial anti-edematous pharmacological therapy, and in selected cases, after radiotherapy.

The goal of surgery is to arrest preoperative neurologic function deterioration and improve the patient’s quality of life through spinal cord decompression. Nevertheless, despite the continuous advances in microsurgical strategies, intraoperative tools, and imaging technologies, this aim is not always achievable. Due to the highly functional and vulnerable nature of the spinal cord, the surgery of intramedullary metastases is a challenge and ranges from biopsy to maximal allowed safe resection. Biopsy is reserved for histological diagnosis or confirmation to plan other treatments or for unresectable cases. A “wait and see” strategy, through close clinical ambulatorial and MRI follow-up each year, is indicated for asymptomatic cases incidentally discovered.

Surgery is indicated in patients with symptomatic large lesions after radiation treatment failure, in cases of sudden onset or rapidly progressive neurological deficit, good preoperative KPS score, or systemic disease with a known indolent growth pattern.

## 5. Conclusions

Several factors affect the prognosis of metastatic RCC, including the extent of disease, histology, grading, and clinical factors [65]. Exeresis of the metastasis and other local treatment strategies, such as conventional radiotherapy, stereotactic radiosurgery, and hypo-fractionated RT, can be considered. The gross-total or subtotal surgical removal of spinal metastasis, either intra or extramedullary, allows one to arrest the progression of neurological deficits and obtain pain relief, improving the quality of life and overall survival of the patient. Considering the relative radioresistant nature of the RCC, surgical treatment of the metastasis is a valid option even if a nerve root was to be sacrificed or a tumoral residue that increases the risk of recurrence was left.

## Figures and Tables

**Table 1:** Reported cases of intradural extramedullary (IDEM) spinal metastasis from renal cell carcinoma.

N of Cases	Author, Year	Sex, Age (Yrs)	Latency to Spinal Metastasis (Months)	Spinal Level	Symptoms	Systemic Metastases	Management of Spinal Metastases	Clinical Outcome	Survival (Months)
1	Takahashi et al. [7]. 1990	M, 51	Simultaneous	L4	LBP, sciatica	n.s.	S, CHT, RT	Decreased L4 sensitivity	Dead at 1
2	Maxwell et al. [8]. 1999	M, 84	60	L2-L3	LBP, sciatica	Lung	S (snr)	Improved	n.s.
3	Mak et al. [9]. 2001	M, 59	48	L3-L4	Weakness, urinary incontinence	Bone	S (pnr)	Improved	Alive at 20
4	Kubota et al. [10]. 2003	M, 68	84	L3	LBP	Lung	S (snr)	Improved	Alive at 24
5	Takada et al. [11]. 2003	M, 61	60	L3	LBP, sciatica, weakness, urinary incontinence	Lung	S (snr), IFN	Motor worsening	Alive at 12
6	Gaetani et al. [12]. 2004	F, 36	4	L3-L4	LBP, sciatica, weakness, urinary incontinence	lepto-meningeal	S, RT	Improved	Dead at 12
7	Alfieri et al. [13]. 2005	F, 67	24	L3-L5	LBP, weakness, urinary symptoms	No	S	Improved	n.s.
8	Jost et al. [14]. 2009	M, 82	6	C6-C7	Left hemiparesis	Brain	S (pnr), RT	Improved	Alive at 12
9	Kim et al. [15]. 2009	M, 41	12	L2	LBP, bilateral leg pain	Lung	S (snr), CHT	Improved	Alive at 12
10	Lin et al. [16]. 2011	M, 68	72	T12-L1	LBP, bilateral leg weakness and sciatica	No	S (pnr), RT, IFN	Improved	Alive at 36
11	Dobson et al. [17]. 2013	F, 81	Simultaneous	L2	LBP, bilateral sciatica, urinary incontinence	No	S (snr), RT	Improved	Alive at 36
12	Ji et al. [18]. 2013	M, 68	192	T12-L1	LBP, bilateral sciatica	Tibia	S (snr), RT	Improved	Alive at 24
13	Strong et al. [19]. 2013	F, 49	96	L4	Left leg weakness and hypoesthesia	No	S (pnr), RT	Improved	Alive at 24
14		M, 72	Simultaneous	L2	asymptomatic	No	S (pnr), RT	Improved	Alive at 24
15	Srinivasan et al. [20]. 2014	M, 40	Simultaneous	L4-S1	Sensory-motor deficits of both legs, urinary incontinence	No	S (pnr)	Improved	Dead during CHT
16	Capek et al. [23]. 2016	F, 61	192	T12	LBP	No	S (snr), RT	Stable	Alive 108
17	Ali et al. [22]. 2021	F, 55	96	L3-L4	LBP	No	S (snr), CHT	Stable	n.s.
18	Madhavan et al. [24]. 2021	M, 68	Simultaneous	T11	acute lower extremities weakness, urinary retention, severe back pain	Lung, lymph-node, bone	CHT (refused S)	Improved	n.s.
19	Mariniello et al. [6]. 2022	M, 64	168	L1-L2, L4-L5	Sudden LBP and left sciatica	Lung	S (pnr), CHT, RT	Improved	Alive at 12

M: Male; F: Female; S: Surgery; RT: Radiotherapy; CHT: Chemotherapy; Med: Medical; IFN: Interferon; LBP: Low Back Pain; yrs: year; pnr: preserved nerve root; snr: sacrified nerve root; n.s.: not specified.

**Table 2:** Demographic, clinical, pathological, management and outcome data of 51 cases of spinal intradural metastasis from RCC.

Covariates	Ovearll Series IDEM Metastases	Ovearll Series ISC Metastases	Statistical Analysis *p* Value
Age (years)	Mean 61.84 ± 16.27 SD (range 36–84 yrs)	Mean 55.96 ± 10.89 SD (range 37–78 yrs)	
Sex			
- Male	13 (68%)	25 (78%)	
- Female	6 (32%)	7 (22%)	
Interval between diagnoses of RCC and IDEM (months)	Mean 61.88 ± 65.65 SD (range 0–192 months)	Mean 28.76 ± 45.31 SD (range 0–180 months)	
- Metachronous	14/19 (74%)	23 (72%)	*p* = 0.889
- Synchronous	5/19 (26%)	9 (28%)	
Spinal level of metastasis - Cervical - Thoracic - Lumbar - Thoraco-lumbar	1/19 (5%) 2/19 (10%) 14/19 (75%) 2/19 (10%)	14/32 (44%) 13/32 (41%) 3/32 (9%) 2/32 (6%)	*p* = 0.0035 *p* = 0.02 *p* < 0.00001 *p* = 0.58
Presenting symptoms - Spinal pain - Radicular symptoms - Leg weakness - Urinary disfunction - Asymptomatic - Motility disturbance - Brown-Sequard syndrome	13/19 (68%) 9/19 (47%) 7/19 (37%) 7/19 (37%) 1/19 (5%) --- ---	8/32 (25%) --- 12/32 (37,5%) 6/32 (19%) --- 2/32 (6%) 2/32 (6%)	*p* = 0.002 *p* = 0.96 *p* = 0.15
Systemic metastases - Lung - Brain - Bone - Leptomeninges - Lymph-nodes - Adrenal gland - Liver - IDEM alone - IM alone	18 * 6/19 (32%) 1/19 (5%) 3/19 (16%) 1/19 (5%) 1/19 (5%) --- --- 8/19 (42%) ---	30 * 15/30 (50%) 12/30 (40%) 6/30 (20%) --- 5/30 (17%) 3/30 (10%) 2/30 (7%) --- 5/30 (17%)	*p* = 0.2 *p* = 0.008 *p* = 1 *p* = 0.3 Single vs. Multiple *p* = 0.036
Management - Surgery - Adjuvant radiotherapy - Adjuvant chemotherapy - Adjuvant IFN therapy - Adjuvant radio- and chemotherapy - Chemotherapy alone - Radiotherapy alone - Pharmacological alone	18/19 (95%) 8/19 (42%) 2/19 (10%) 2/19 (10%) 2/19 (10%) 1/19 (5%) ---	11/32 (35%) 10/32 (31%) 9/32 (28%) 2/32 (6%)	*p* < 0.0001 *p* = 0.68
Clinical outcome - Improved - Stable - Worsened	15/19 (80%) 2/19 (10%) 2/19 (10%)	27 * 16/27 (59%) 9/27 (34%) 2/27 (7%)	*p* = 0.16 *p* = 0.09 *p* = 0.71
Survival (months) - Alive at last follow-up - Dead at last follow-up	15 * Mean 25.5 ± 25.6 SD (range 1–108 months) 12/15 (80%) 3/15 (20%)	28 * Mean 13.15 ± 17.09 SD (range 1–65 months) 14/28 (50%) 14/28 (50%)	*p* = 0.011

* Cases with reported data; IDEM, intradural extramedullary; ISC intramedullary spinal cord.

**Table 3:** Reported cases of intramedullary spinal cord metastasis from renal cell carcinoma.

*n* of Cases	Author, Year	Sex, Age (yrs)	Latency to Spinal Metastasis (Months)	Spinal Level	Symptoms	Systemic Metastases	Management of Spinal Metastases	Clinical Outcome	Survival (Months)
1	Ateaque et al. [25]. 2000	M, 63	132	C2-C3	Ataxia, tetraparesis	No	S	Stable	Dead at 1
2	Schijns et al. [26]. 2000	F, 70	Simultaneous	C7	Cervical-brachialgia, paraparesis	Liver	S	Improved	Alive at 13
3	Fakih et al. [27]. 2001	M, 56	0	C4	Lower extremities weakness, urinary incontinence	Brain, lung	Med, RT	Improved	Dead at 6
4		M, 60	180	T1-T2	Lower extremity weakness	Brain, lung	S, RT	Improved	Alive at 5
5		F, 68	2	T8-L2	Lower extremities weakness	No	Med, RT	Improved	Dead at 11
6		F, 57	Simultaneous	C7	Brown-Sequard syndrome	Brain, lung	Med, RT	Improved	Dead at 6
7		M, 46	2	T5	Leg weakness, urinary disfunction	Brain, lung, lymph-nodes	RT, Med	Stable	Dead at 4
8		F, 37	25	C2	Bilateral cervical-brachialgia	Lung	S	Improved	Dead at 23
9	Poggi et al. [28]. 2001	M, 37	2	T12	Dysesthesia right leg	Brain, bone, lymph-nodes	RT, Med	n.s.	n.s
10	Kaya et al. [29]. 2003	M, 43	12	L1	LBP, urinary incontinence, lower extremities weakness	n.s.	S	Improved	Dead at 6
11	Altinoz et al. [30]. 2005	M, 43	26	T6-T7	Back pain, leg weakness	Brain, lung, adrenal gland	S	Stable	Alive at 25
12	Gomez de la Riva et al. [31]. 2005	M, 69	Simultaneous	L1	Lower extremities weakness	Lung	S	Improved	Alive at 16
13	Donovan et al. [32]. 2006	F, 41	Simultaneous	C4	Brown-Sequard syndrome	Lung, bone	S, RT	Worsened	Dead at 2
14	Asadi et al. [33]. 2009	F, 51	Simultaneous	T12	Back pain, paraparesis	Brain, bone	palliative	n.s.	n.s
15	Parikh et al. [34]. 2009	M, 50	4	C5	Upper extremities paresthesiae	Brain, lymph-nodes	RT, SRS	Stable	Alive at 26
16	Petrelli et al. [35]. 2010	F, 57	Simultaneous	T12-L1	Paraparesis, paresthesia, hypoesthesia	Lung, bone, lymph-nodes	CHT, RT	Improved	Alive at 6
17	Komura et al. [36]. 2011	M, 57	60	C4	Bilateral shoulder pain, upper and lower extremities weakness	No	S	Improved	Alive at 22
18	Zakaria et al. [37]. 2012	M, 62	2	C7	Back pain, urinary incontinence, lower limb weakness	Lung, lymph-nodes	RT, S, Med	Improved	Dead at 3
19	Park et al. [38]. 2013	M, 44	6	T12	Paraparesis	Lung	RT, S	Improved	Alive at 6
20	Gao et al. [39]. 2014	M, 51	72	T4-T5	Lower extremities weakness, urinary incontinence	No	S	Improved	Alive at 3
21	Nomoto et al. [40]. 2016	M 48	5	T8-T9	Paraplegia	Lung	RT, S	n.s.	Alive at 3
22	Soga et al. [41]. 2016	M 69	3	T12	Paraplegia, urinary retention	Lung	Refused S, Med	Worsened	Dead at 3
23	Islam et al. [42]. 2016	M 62	Simultaneous	T12	Spastic paraparesis	Bone	Refused S, RT	Improved	Alive at 1
24	Weng et al. [43]. 2018	M 58	34	T12	Lower extremities numbness, paraparesis	Lung	S, RT	Improved	Alive at 6
25	Malik et al. [44]. 2018	M 75	Simultaneous	T11-T12	Lower extremity weakness	n.s.	S, RT	n.s.	n.s.
26	Strickland et al. [45]. 2018	M 50	64	C1	n.s.	Brain	S	Stable	Dead at 6.5
27		M 50	92	C5	n.s.	Brain	S, RT	Stable	Dead at 2.9
28		M 66	97	T11	n.s.	Bone	S, RT	Stable	Alive at 65
29		M 59	32	C3	n.s.	Brain	S	Stable	Alive at 65
30	Barrie et al. [46]. 2019	M 56	5	C2-C3	Left facial weakness, diplopia, left upper and lower extremity weakness	Brain, adrenal gland, lung, liver, mediastinum	RT, CHT, Med, S	Stable	Dead at 1
31	Ponzo et al. [47]. 2020	M, 78	Simultaneous	C1-C2	Cervicalgia, hemiplegia	Muscle, adrenal gland	S	Improved	Dead at 14
32	Kalimuthu et al. [48]. 2020	M, 65	6	L1-L2	LBP	No	RT	n.s.	n.s.

M: Male; F: Female; S: Surgery; RT: Radiotherapy; CHT: Chemotherapy; Med: Medical; LBP: Low Back Pain.

**Table 4:** Outcome and overall survival data according to the treatment in 19 intradural extramedullary spinal metastases from RCC.

Treatment	Clinical Outcome				Overall Survival		
	Stable	Improved	Worsened	n.s.	Alive (Months)	Dead	n.s.
Surgery alone (5/19)	0	5	0	0	2 (20–24 mo.) (Mean 22 ± 2.8 SD mo.)	1 (Mean 22 ± 2.8 SD mo.)	2
Adjuvant radiotherapy (8/19)	1	7	0	0	7 (12–108 mo.) (Mean 32 ± 37.7 SD mo.)	1 (12 mo.)	0
Adjuvant chemotherapy (2/19)	1	1	0	0	1 (12 mo.)	0	1
Adjuvant IFN therapy (2/19)	0	1	1	0	2 (12–36 mo.) (Mean 24 ± 16.9 SD mo.)	0	0
Adjuvant radio and chemotherapy (2/19)	0	1	1	0	1 (12 mo.)	1 (1 mo.)	0
Chemotherapy alone (1/19)	0	1	0	0	0	0	1

n.s.: not specified, mo.: months.

**Table 5:** Outcome and overall survival data according to the treatment in 32 intramedullary spinal cord metastases from RCC.

Treatment	Clinical Outcome				Overall Survival		
	Stable	Improved	Worsened	n.s.	Alive	Dead	n.s.
Surgery alone (11/32)	4	7	0	0	6 (3–65 mo.) (Mean 24 ± 21.5 SD mo.)	5 (1–23 mo.) (Mean 11 ± 9.6 SD mo.)	0
Radiotherapy alone (9/32)	2	5	0	2	3 (1–26 mo.) (Mean 11 ± 13.22 SD mo.)	4 (4–11 mo.) (Mean 6.75 ± 2.98 SD mo.)	2
Surgery + Radiotherapy (10/32)	3	4	1	2	5 (3–65 mo.) (Mean 17 ± 26.8 SD mo.)	4 (1–3 mo.) (Mean 2 ± 1 SD mo.)	1

n.s.: not specified, mo.: months.

## References

[1] Saad A.M., Gad M.M., Al-Husseini M.J., Ruhban I.A., Sonbol M.B., Ho T.H. (2019). Trends in Renal-Cell Carcinoma Incidence and Mortality in the United States in the Last 2 Decades: A SEER-Based Study. Clin. Genitourin. Cancer.

[2] Choueiri T.K., Motzer R.J. (2017). Systemic Therapy for Metastatic Renal-Cell Carcinoma. N. Engl. J. Med..

[3] Brown L.C., Desai K., Zhang T., Ornstein M.C. (2020). The Immunotherapy Landscape in Renal Cell Carcinoma. BioDrugs.

[4] Dabestani S., Thorstenson A., Lindblad P., Harmenberg U., Ljungberg B., Lundstam S. (2016). Renal cell carcinoma recurrences and metastases in primary non-metastatic patients: A population-based study. World J. Urol..

[5] Bianchi M., Sun M., Jeldres C., Shariat S.F., Trinh Q.D., Briganti A., Tian Z., Schmitges J., Graefen M., Perrotte P. (2012). Distribution of metastatic sites in renal cell carcinoma: A population-based analysis. Ann. Oncol..

[6] Mariniello G., Corvino S., Sgulò F., Guadagno G., Del Basso De Caro M., Maiuri F. (2022). Intradural cauda equina metastases from renal cell carcinoma. Interdiscip. Neurosurg.

[7] Takahashi I., Isu T., Iwasaki Y., Akino M., Takahashi A., Abe H., Kitagawa M., Kojima H., Inoue K., Saitoh H. (1990). Metastatic Grawitz’s tumor to the cauda equina: Case report. No Shinkei Geka.

[8] Maxwell M., Borges L.F., Zervas N.T. (1999). Renal cell carcinoma: A rare source of cauda equina metastasis. Case report. J. Neurosurg..

[9] Mak K.H., Kwok J.C. (2001). Intradural spinal metastasis from renal cell carcinoma: A case report. J. Orthop. Surg..

[10] Kubota M., Saeki N., Yamaura A., Iuchi T., Ohga M., Osato K. (2004). A rare case of metastatic renal cell carcinoma resembling a nerve sheath tumor of the cauda equina. J. Clin. Neurosci..

[11] Takada T., Doita M., Nishida K., Miura J., Yoshiya S., Kurosaka M. (2003). Unusual metastasis to the cauda equina from renal cell carcinoma. Spine.

[12] Gaetani P., Di Ieva A., Colombo P., Tancioni F., Aimar E., Debernardi A., Rodriguez Y., Baena R. (2004). Intradural spinal metastasis of renal clear cell carcinoma causing cauda equina syndrome. Acta Neurochir..

[13] Alfieri A., Mazzoleni G., Schwarz A., Campello M., Broger M., Vitale M., Vigl E.E. (2005). Renal cell carcinoma and intradural spinal metastasis with cauda equina infiltration: Case report. Spine.

[14] Jost G., Zimmerer S., Frank S., Cordier D., Merlo A. (2009). Intradural spinal metastasis of renal cell cancer. Report of a case and review of 26 published cases. Acta Neurochir..

[15] Kim D.Y., Lee J.K., Moon S.J., Kim S.C., Kim C.S. (2009). Intradural spinal metastasis to the cauda equina in renal cell carcinoma: A case report and review of the literature. Spine.

[16] Lin T.K., Chen S.M., Jung S.M. (2011). Solitary intradural extramedullary metastasis of renal cell carcinoma to the conus medullaris. Kaohsiung J. Med. Sci..

[17] Dobson G.M., Polvikoski T., Nissen J.J., Holliman D. (2013). Cauda equina syndrome secondary to intradural renal cell carcinoma metastasis haemorrhage. Br. J. Neurosurg..

[18] Ji G.Y., Oh C.H., Kim S.H., Shin D.A., Kim K.N. (2013). Intradural cauda equina metastasis of renal cell carcinoma: A case report with literature review of 10 cases. Spine.

[19] Strong C., Yanamadala V., Khanna A., Walcott B.P., Nahed B.V., Borges L.F., Coumans J.V. (2013). Surgical treatment options and management strategies of metastatic renal cell carcinoma to the lumbar spinal nerve roots. J. Clin. Neurosci..

[20] Srinivasan A., Dhandapani S., Chatterjee D., Simha V. (2014). Renal small cell carcinoma presenting with solitary lumbar intradural metastasis. Neurol. India.

[21] Carminucci A., Hanft S. (2020). Intradural extramedullary spinal metastasis of renal cell carcinoma: Illustrative case report and comprehensive review of the literature. Eur. Spine J..

[22] Ali S., Qasim A., Salah R., Sarwar M.R., Usman M., Shams S. (2021). Isolated late intradural cauda equina metastasis of renal cell carcinoma. Surg. Neurol. Int..

[23] Capek S., Krauss W.E., Amrami K.K., Parisi J.E., Spinner R.J. (2016). Perineural Spread of Renal Cell Carcinoma: A Case Illustration with a Proposed Anatomic Mechanism and a Review of the Literature. World Neurosurg..

[24] Madhavan A.A., Eckel L.J., Carr C.M., Diehn F.E., Lehman V.T. (2021). Subdural spinal metastases detected on CT myelography: A case report and brief review. Radiol. Case Rep..

[25] Ateaque A., Martin J.L., O’Brien C. (2000). Intramedullary spinal cord metastases from a hypernephroma 11 years following the diagnosis and treatment of the primary lesion. Br. J. Neurosurg..

[26] Schijns O.E., Kurt E., Wessels P., Luijckx G.J., Beuls E.A. (2000). Intramedullary spinal cord metastasis as a first manifestation of a renal cell carcinoma: Report of a case and review of the literature. Clin. Neurol. Neurosurg..

[27] Fakih M., Schiff D., Erlich R., Logan T.F. (2001). Intramedullary spinal cord metastasis (ISCM) in renal cell carcinoma: A series of six cases. Ann. Oncol..

[28] Poggi M.M., Patronas N., Buttman J.A., Hewitt S.M., Fuller B. (2001). Intramedullary spinal cord metastasis from renal cell carcinoma: Detection by positron emission tomography. Clin. Nucl. Med..

[29] Kaya R.A., Dalkiliç T., Ozer F., Aydin Y. (2003). Intramedullary spinal cord metastasis: A rare and devastating complication of cancer—Two case reports. Neurol. Med. Chir.

[30] Altinoz M.A., Santaguida C., Guiot M.C., Del Maestro R.F. (2005). Spinal hemangioblastoma containing metastatic renal cell carcinoma in von Hippel-Lindau disease. Case report and review of the literature. J. Neurosurg. Spine.

[31] Gómez de la Riva A., Isla A., Pérez-López C., Budke M., Gutiérrez M., Frutos R. (2005). Intramedullary spinal cord metastasis as the first manifestation of a renal carcinoma. Neurocirugia.

[32] Donovan D.J., Freeman J.H. (2006). Solitary intramedullary spinal cord tumor presenting as the initial manifestation of metastatic renal cell carcinoma: Case report. Spine.

[33] Asadi M., Rokni-Yazdi H., Salehinia F., Allameh F.S. (2009). Metastatic renal cell carcinoma initially presented with an intramedullary spinal cord lesion: A case report. Cases J..

[34] Parikh S., Heron D.E. (2009). Fractionated radiosurgical management of intramedullary spinal cord metastasis: A case report and review of the literature. Clin. Neurol. Neurosurg..

[35] Petrelli F., Cabiddu M., Carpo M., Ghilardi M., Barni S. (2010). Progression of intramedullary metastasis during perioperative cessation of sunitinib. Nat. Rev. Urol..

[36] Komura S., Myamoto K., Hosoe H., Iwata A. (2011). Intramedullary spinal cord metastasis from renal cell carcinoma mimicking intramedullary hemangioblastoma. Eur. J. Orthop. Surg. Traumatol..

[37] Zakaria Z., Fenton E., Jansen M., O’Brien D. (2012). The occult nature of intramedullary spinal cord metastases from renal cell carcinoma. BMJ Case Rep..

[38] Park J., Chung S.W., Kim K.T., Cho D.C., Hwang J.H., Sung J.K., Lee D. (2013). Intramedullary spinal cord metastasis in renal cell carcinoma: A case report of the surgical experience. J. Korean Med. Sci..

[39] Gao J., Li Y., Yang Z., Wang R. (2014). Intramedullary spinal cord metastasis of renal cell carcinoma 6 years following the nephrectomy. Turk. Neurosurg..

[40] Nomoto Y., Tsukie T., Kurita A., Seki K., Suzuki H., Yamazaki K. (2016). Metastatic renal cell carcinoma initially presented with a longitudinally extensive spinal cord lesion on MRI. Rinsho Shinkeigaku.

[41] Soga H., Imanishi O. (2016). Case of intramedullary spinal cord metastasis of renal cell carcinoma. World J. Clin. Urol..

[42] Islam R. (2016). Renal Cell Carcinoma presented with An Intramedullary Spinal Cord Metastasis: A Case Report. Bangladesh Crit. Care J..

[43] Weng Y., Zhan R., Shen J., Pan J., Jiang H., Huang K., Xu K., Huang H. (2018). Intramedullary Spinal Cord Metastasis from Renal Cell Carcinoma: A Systematic Review of the Literature. Biomed. Res. Int.

[44] Malik M.T., Kazmi S.J., Turner S. (2018). Teaching NeuroImages: Intradural, intramedullary spinal cord metastasis from primary renal cell carcinoma. Neurology.

[45] Strickland B.A., McCutcheon I.E., Chakrabarti I., Rhines L.D., Weinberg J.S. (2018). The surgical treatment of metastatic spine tumors within the intramedullary compartment. J. Neurosurg. Spine.

[46] Barrie U., Elguindy M., Pernik M., Adeyemo E., Aoun S.G., Hall K., Reyes V.P., El Ahmadieh T.Y., Bagley C.A. (2020). Intramedullary Spinal Metastatic Renal Cell Carcinoma: Systematic Review of Disease Presentation, Treatment, and Prognosis with Case Illustration. World Neurosurg..

[47] Ponzo G., Umana G.E., Giuffrida M., Furnari M., Nicoletti G.F., Scalia G. (2020). Intramedullary craniovertebral junction metastasis leading to the diagnosis of underlying renal cell carcinoma. Surg. Neurol. Int..

[48] Kalimuthu L.M., Ora M., Gambhir S. (2020). Recurrent Renal Carcinoma with Solitary Intramedullary Spinal Cord Metastasis. Indian J. Nucl. Med..

[49] Mosdal C., Bang F. (1981). Intradural spinal metastases. Acta Neurochir..

[50] Westphal M., Mende K.C., Eicker S.O. (2021). Refining the treatment of spinal cord lesions: Experience from 500 cases. Neurosurg. Focus.

[51] Cofano F., Giambra C., Costa P., Zeppa P., Bianconi A., Mammi M., Monticelli M., Di Perna G., Junemann C.V., Melcarne A. (2020). Management of Extramedullary Intradural Spinal Tumors: The Impact of Clinical Status, Intraoperative Neurophysiological Monitoring and Surgical Approach on Outcomes in a 12-Year Double-Center Experience. Front. Neurol..

[52] Ahn A., Phan K., Cheung Z.B., White S.J.W., Kim J.S., Cho S.K. (2019). Predictors of Discharge Disposition Following Laminectomy for Intradural Extramedullary Spinal Tumors. World Neurosurg..

[53] Tang C., Msaouel P., Hara K., Choi H., Le V., Shah A.Y., Wang J., Jonasch E., Choi S., Nguyen Q.N. (2021). Definitive radiotherapy in lieu of systemic therapy for oligometastatic renal cell carcinoma: A single-arm, single-centre, feasibility, phase 2 trial. Lancet Oncol..

[54] Kjaer M., Frederiksen P.L., Engelholm S.A. (1987). Postoperative radiotherapy in stage II and III renal adenocarcinoma. A randomized trial by the Copenhagen Renal Cancer Study Group. Int J. Radiat. Oncol. Biol. Phys..

[55] Deschavanne P.J., Fertil B. (1996). A review of human cell radiosensitivity in vitro. Int. J. Radiat. Oncol. Biol. Phys..

[56] Dabestani S., Marconi L., Hofmann F., Stewart F., Lam T.B., Canfield S.E., Staehler M., Powles T., Ljungberg B., Bex A. (2014). Local treatments for metastases of renal cell carcinoma: A systematic review. Lancet Oncol..

[57] Alt A.L., Boorjian S.A., Lohse C.M., Costello B.A., Leibovich B.C., Blute M.L. (2011). Survival after complete surgical resection of multiple metastases from renal cell carcinoma. Cancer.

[58] Kwak C., Park Y.H., Jeong C.W., Lee S.E., Ku J.H. (2007). Metastasectomy without systemic therapy in metastatic renal cell carcinoma: Comparison with conservative treatment. Urol. Int..

[59] Ouzaid I., Capitanio U., Staehler M., Wood C.G., Leibovich B.C., Ljungberg B., Van Poppel H., Bensalah K., Young Academic Urologists Kidney Cancer Working Group of the European Association of Urology (2019). Surgical Metastasectomy in Renal Cell Carcinoma: A Systematic Review. Eur. Urol. Oncol..

[60] Khoo V.S., Pyle L., Eisen T., Christmas T. (2007). Radiotherapy and Supportive Care.

[61] Maor M.H., Frias A.E., Oswald M.J. (1988). Palliative radiotherapy for brain metastases in renal carcinoma. Cancer.

[62] Ljungberg B., Bensalah K., Canfield S., Dabestani S., Hofmann F., Hora M., Kuczyk M.A., Lam T., Marconi L., Merseburger A.S. (2015). EAU guidelines on renal cell carcinoma: 2014 update. Eur. Urol..

[63] Samartzis D., Gillis C.C., Shih P., O’Toole J.E., Fessler R.G. (2015). Intramedullary Spinal Cord Tumors: Part I-Epidemiology, Pathophysiology, and Diagnosis. Global Spine J..

[64] Payer S., Mende K.C., Westphal M., Eicker S.O. (2015). Intramedullary spinal cord metastases: An increasingly common diagnosis. Neurosurg. Focus.

[65] Moch H., Cubilla A.L., Humphrey P.A., Reuter V.E., Ulbright T.M. (2016). The 2016 WHO Classification of Tumours of the Urinary System and Male Genital Organs-Part A: Renal, Penile, and Testicular Tumours. Eur. Urol..

